# Comparison of the urgent referral for suspected breast cancer process with patient age and a predictive multivariable model

**DOI:** 10.1093/bjsopen/zraa023

**Published:** 2020-12-23

**Authors:** S Ramzi, P J Cant

**Affiliations:** Primrose Breast Care Centre, Derriford Hospital, University Hospitals Plymouth NHS Trust, UK

## Abstract

**Background:**

The urgent 2-week wait referral for suspected breast cancer system (U2WW) in the UK prioritizes primary care referrals to one-stop breast clinics as ‘urgent’ or ‘choose and book’ (C&B). The aim of this study was to evaluate the accuracy of U2WW in discriminating cancer *versus* no cancer, and to consider alternative criteria.

**Methods:**

Clinical features elicited in primary care and demographics of consecutive female patients in a specialist breast clinic were collated at the time of consultation from May 2008 to July 2017. U2WW was compared with patient age alone and a multivariable model in terms of accuracy and net cost for eight underlying cost–benefit assumptions.

**Results:**

There were 7915 eligible referrals: 4877 urgent (61.6 per cent) and 3038 C&B (38.4 per cent) referrals. Breast cancer was diagnosed in 546 patients (6.9 per cent): 491 (10.1 per cent) in urgent and 55 (1.8 per cent) in C&B referrals *(P < *0.001). The multivariable model summated the significant variables: age (odds ratio (OR) 1.07, 95 per cent c.i. 1.07 to 1.08), tumour (OR 4.85, 3.62 to 6.52), observed change (OR 1.73, 1.34 to 2.23), pain (OR 0.46, 0.35 to 0.61) and gravidity (OR 0.72, 0.54 to 0.95). The area under the curve was 0.651 for U2WW, 0.784 for age alone, and 0.824 for the multivariable model (*P* <0.001 for all comparisons). Considering the cost assumptions, age alone and the multivariable model were either more accurate than U2WW, or as accurate but less costly.

**Conclusion:**

The U2WW is surpassed by patient age as a single referral criterion. A multivariable model based on demographics and simple clinical features outperformed both. The continued use of the U2WW needs to be reconsidered.

## Introduction

Worldwide, breast cancer is the second commonest cancer^1^, and in the UK 54 700 women are affected annually^2^, of whom 60 per cent present symptomatically to specialist breast clinics^3^^,^^4^. Breast cancer is the second most common cause of cancer death in the UK, with around 11 400 deaths per year^5^.

Early diagnosis improves cancer outcomes by providing care at the earliest possible stage^6^. Breast screening of asymptomatic women reduces the mortality associated with breast cancer through early diagnosis during the symptom-free preclinical phase^7^, and early diagnosis resulting from the prompt referral of patients presenting with early symptoms to the appropriate venue of opinion and investigations improves outcomes^6^^,^^8^.

Like many other countries (such as Australia, Canada and many European countries), primary care practice in the UK serves as the symptomatic patient’s first point of entry into the healthcare system (gatekeeping) and patients do not normally have direct access to hospital consultants^9^. In primary care, patients are evaluated mostly by general practitioners (GPs) by a clinical process, essentially history and examination, as special breast investigations (breast imaging with or without biopsy) are not available to them directly and can be offered only in secondary care by breast specialists. Subsequently, GPs make decisions as to whether referral to specialist breast clinic is required, mostly owing to suspicion of cancer. According to the WHO, late-stage presentation of cancer is still common and improved referral from primary to secondary care is essential for early diagnosis. The WHO recognizes suboptimal knowledge at the primary care level regarding cancer symptoms, unclear referral pathways, and poor accessibility as barriers to early diagnosis^1^. In the UK, more than 1 in 10 breast cancer cases are diagnosed late^2^.

The breast cancer risk evaluation and access are controlled by Public Health England using the urgent 2-week referral for suspected breast cancer system (U2WW)^10^, part of the ‘Referral to Treatment Target’ that is required and recorded for the National Health Service^11^. The U2WW is represented by a list of mutually independent clinical criteria. Patients who fulfil any of the U2WW criteria (*Table 1*) are referred from primary to secondary care urgently, and those who do not are referred through the ‘choose & book’ (C&B) route. The rule to appoint patients into a specialist breast clinic within 2 weeks of receiving an urgent primary care referral was introduced in 1998, with the aiming of reducing the preclinical phase of breast cancer and save lives^12^.

**Table 1:** Urgent 2-week referral for suspected breast cancer criteria

Before November 2016
Women of any age: A fixed hard lump Age over 30 years with discrete mass persisting after next period or presenting after the menopause New lump in women with previous breast carcinoma (including carcinoma *in situ*) Unilateral eczematous skin or nipple change not responding to topical treatment Nipple distortion of recent onset Spontaneous unilateral bloody or clear nipple discharge Skin tethering or dimpling Women aged under 30 years Enlarging lump A lump that is fixed and hard
**After November 2016**
Refer people using a suspected cancer pathway referral (for appointment within 2 weeks) for breast cancer if they are: aged 30 years or above and have an unexplained breast lump with or without pain; or aged 50 years and over with any of the following symptoms in one nipple only: discharge retraction other changes of concern Consider a suspected cancer pathway referral (for appointment within 2 weeks) for breast cancer in people: with skin changes that suggest breast cancer aged 30 years and over with an unexplained lump in the axilla Consider non-urgent referral in people aged under 30 years with an unexplained breast lump with or without pain

However, early published evaluations of the U2WW were not positive^13^^,^^14^. Variable adherence to guidelines increased inappropriate referrals considerably^15^. Furthermore, the U2WW produced an increase in the number of urgent referrals over time with decreasing positive predictive value (PPV), but, more importantly, there was an increase in the waiting times for patients with breast cancer who were not referred by the urgent route^16^^,^^17^. Consequently, the 2-week rule was changed in 2010 to include all new breast referrals (C&B as well as urgent)^11^, defeating the purpose of two distinct referral routes.

Furthermore, in June 2015, the referral criteria were modified (*Table 1*), so that they corresponded to a 3 per cent or greater risk of breast cancer (5 per cent previously)^10^. This was intended to improve the sensitivity of the process and subsequently reduce the rate at which breast cancer was diagnosed among women referred via C&B, the false omission rate (FOR).

A highly accurate test at discriminating cancer *versus* no cancer in primary care allows safe reallocation of resources in secondary breast care. It also allows the adoption of different time mandates for appointing patients to specialist clinics; high-risk patients are seen sooner in triple-assessment one-stop clinics, and low-risk patients are safely deferred and/or seen in less resourced clinics. As there has been no recent evaluation of the U2WW criteria to justify its continued use, the aim of this study is to evaluate the performance of the U2WW referral process and assess alternatives.

## Methods

The study was granted ethical approval from the Health Research Authority and Health and Care Research Wales (reference 19/LO/1737). Consecutive referrals of female patients seen by a consultant breast surgeon at a specialist breast clinic constituted the study population. Data regarding the type of referral (urgent *versus* C&B), patient demographics, and the clinical features (signs and symptoms) elicited in primary care were used to populate a clinical database at the time of consultation.

The outcome of the consultation (cancer *versus* no cancer) was recorded subsequently. Breast cancer was defined as any malignant lesion of the breast: invasive or ductal carcinoma *in situ*. Multiple new referrals of the same patient were included as unique records, and there was no age restriction. Referrals of male patients, secondary and tertiary care referrals, and follow-up consultations that were not associated with a new primary care referral were excluded.

### Clinical features

Data on signs and symptoms were collated from primary care referral documents. The referrer usually indicated the patient’s presenting features by ticking the corresponding criterion on the urgent referral form. Additional clinical features were also extracted from the free-text part, if present, of urgent and C&B referrals. For this study, clinical features were stratified into three clinically relevant categories (tumour (T), observation (O) and somatosensory perception (P): TOP-T, TOP-O and TOP-P), which were not mutually exclusive (*Table 2*).

**Table 2:** Definitions of the variables included in statistical analysis

Patient demographics
Age at date of clinic consultation Personal history of breast cancer, including *in situ* malignancy Menopause: an approximation based on amenorrhoea and age. Premenopausal, menstruation of any frequency *versus* postmenopausal due to natural progression or intervention Gravidity: previous pregnancy of any frequency or duration Breastfeeding of any attempt or duration HRT: any attempt, type, topical or systemic, current or previous, and for any duration Family history stratified as: First degree: if at least one first-degree relative had breast or ovarian cancer at any age Second degree: if at least one second-degree relative had breast or ovarian cancer, and first-degree relatives were unaffected No or other: if none or only third-degree relatives were affected
**Clinical features as reported in primary care urgent or C&B referrals (acronym TOP)**
Tumour (T): any reference to an abnormality detected by primary healthcare professionals by palpation; includes breast or axillary lump, thickening, nodularity, firmness, hardness, lumps in the axilla or supraclavicular Observation (O): any reference to something visual, seen on inspection by primary healthcare professionals; includes: Nipple signs: blood or serous discharge, inversion, flattening, creasing, retraction, shrinkage Skin signs: tethering, dimpling, puckering, skin thickening, oedema, redness, discoloration, bruising Skin lesion: eczema, cyst, rash, ulcer, abscess, boils, cellulitis Swelling, enlargement, visible asymmetry, distortion, shrinkage Somatosensory perception (P): any reference to subjective patient sensation, usually pain; includes pain, tenderness, awareness, itch, discomfort, sensitivity, heaviness, tingling, burning

HRT, hormone replacement therapy; C&B, choose and book.

### Statistical analysis

The study compared three referral models: U2WW, age alone, and a multivariable model that summated significant demographic and clinical features (TOP terms). Statistical analysis was performed using MedCalc^®^ statistical software version 19.1 (MedCalc Software, Ostend, Belgium; https://www.medcalc.org). The level of significance was set at *P* < 0.050 (two-tailed). Mann – Whitney *U* and χ^2^ tests were used to analyse continuous and categorical variables respectively. Multivariable analysis was performed using logistic regression. Variables with *P* < 0.250 in univariable logistic regression analysis were included in the multivariable logistic regression analysis, and after stepwise exclusion the final multivariable model included variables with *P* < 0.050 only.

The following accuracy parameters were evaluated: sensitivity (proportion of breast cancer records that were urgent referrals); specificity (proportion of non-cancer records that were C&B referrals); PPV (proportion of urgent referrals with a breast cancer outcome); FOR (proportion of C&B referrals with a breast cancer outcome); and receiver operating characteristic (ROC) curve analysis for paired or independent samples as appropriate, expressed as the area under the curve (AUC).

### Optimal referral threshold

In the context of this study, the optimal referral threshold (ORT) was the cut-off value of the respective model at which the net cost (health plus financial) was minimal^18^. It was calculated by an analysis of the prevalence of breast cancer in the sample and the costs and benefits of the four possible outcomes of the respective referral model: false negative (FNc—the cost of referring a patient with breast cancer through C&B); false positive (FPC—the cost of referring a patient without breast cancer urgently); true positive (TPc—the benefit of referring a patient with breast cancer urgently); and true negative (TNc—the benefit of referring a patient without breast cancer through C&B). Eight ORTs were identified for each of the three models, each corresponding to a cost–benefit assumption. Subsequently, the models were dichotomized at their respective ORTs and compared with regard to net cost and accuracy parameters.

### Rationale for the cost–benefit assumptions

The modification of the referral criteria to lower the threshold for urgent referrals in June 2015^6^ aimed to reduce the proportion of women with breast cancer referred through the routine route (false negatives) even if it resulted in an increase in the proportion of women without breast cancer referred urgently (false positives). Consequently, for the model it was assumed that FNc was more costly than FPc by 50 : 1, 100 : 1 and 150 : 1. Additional support to this assumption is offered by the fact that the 2-week rule was extended to C&B referrals in an effort to mitigate the potential harm to breast cancer patients referred routinely^11^. The models were also compared at FNc : FPc=1 : 1, to demonstrate their behaviour at low relative cost. The appropriate referral of women without cancer routinely (true negatives) and those women with breast cancer urgently (true positives) results in the appropriate allocation of resources, which can be viewed to indicate equal benefit from true-positive decisions. However, true positives appear to be highly valued despite any health benefit being theoretical at best; therefore, two assumptions were made regarding benefit (expressed as negative cost): TPc : TNc = −1 :−1 and −10 :−1.

## Results

The study database included 7915 eligible referrals of 7470 female patients (445 were referred between 2 and 5 times); 4877 (61.6 per cent) were urgent and 3038 (38.4 per cent) were C&B referrals. A diagnosis of breast cancer was made in 546 (6.9 per cent) of the 7915 referrals: 55 in C&B referrals (1.8 per cent) and 491 in urgent referrals (10.1 per cent). Median patient age was 46.0 (range 1–100) years.

### New *versus* old U2WW criteria

**Fig. 1:**
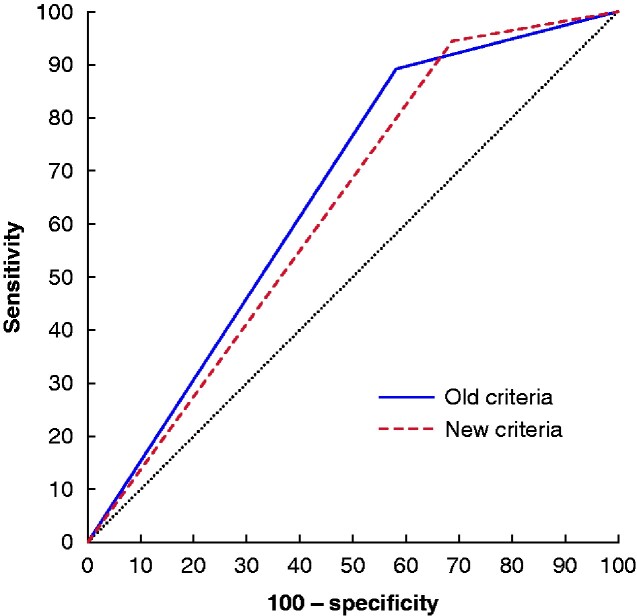
Comparison of receiver operating characteristic (ROC) curves for the old (before June 2015) and new (after June 2015) urgent 2-week referral for suspected breast cancer criteria (U2WW) with respect to discrimination of cancer *versus* no cancer Area under the ROC curve (AUC): 0.656 (95 per cent c.i. 0.644 to 0.667) for old criteria (*n*=6866) *versus* 0.629 (0.599 to 0.658) for new criteria (*n*=1049); *P* =0.116).

Analysis of the accuracy parameters for the current *versus* old U2WW showed that the old U2WW was at least as accurate (*Fig. 1*); therefore, only an analysis of U2WW in the two periods combined is presented. Sensitivity (94.5 *versus* 89.2 per cent for current and old U2WW respectively; *P* = 0.161) and FOR (1.9 *versus* 1.3 per cent; *P* = 0.447) were not significantly different. Specificity, however, was significantly lower with the current version (31.3 per cent *versus* 41.9 per cent for the old version; *P* < 0.001).

### Demographics and clinical features

Univariable and multivariable analyses of patient demographics and clinical features are shown in *Table 3*. The final multivariable model included: gravidity (odds ratio (OR) 0.72, 95 per cent c.i. 0.54 to 0.95; *P* = 0.021) and TOP-P (OR 0.46, 0.35 to 0.61; *P* < 0.001) as negative predictors, and age (OR 1.07, 1.06 to 1.08; *P* < 0.001), TOP-T (OR 4.85, 3.62 to 6.52; *P* < 0.001) and TOP-O (OR 1.73, 1.34 to 2.23; *P* < 0.001) as positive predictors.

**Table 3:** Univariable and multivariable analysis of predictors of breast cancer in primary healthcare

	**All patients** **(*n* = 7915)**	**No cancer** **(*n* = 7369)**	**Cancer** **(*n* = 546)**	Univariable *P*	Multivariable analysis§	
					**Odds ratio** ^*^	*P*
**Demographics**						
Age (years)^†^	46.0 (36.0–57.0)	45,0 (35.0–55,0)	66.0 (49.0–79.0)	<0.001	1.07 (1.07, 1.08)	<0.001
Previous breast cancer				<0.001		
No	7272 (91.9)	6810 (92.4)	462 (84.6)			
Yes	643 (8.1)	559 (7.6)	84 (15.4)			
Menopause				0.002		
No	4804 (60.7)	4514 (61.3)	290 (53.1)			
Yes	3111 (39.3)	2855 (38.7)	256 (46.9)			
Gravidity				0.123		
No	1368 (17.4)	1288 (17.6)	80 (15.0)		1.00 (reference)	
Yes	6497 (82.6)	6042 (82.4)	455 (85.0)		0.72 (0.54, 0.95)	0.021
Missing	50	39	11			
Breastfeeding				0.278		
No	3945 (50.4)	3692 (50.5)	253 (48.1)			
Yes	3885 (49.6)	3612 (49.5)	273 (51.9)			
Missing	85	65	20			
Family history				0.229		
No/other	5257 (67.2)	4910 (67.4)	347 (64.5)			
Second degree	1323 (16.9)	1218 (16.7)	105 (19.5)			
First degree	1247 (15.9)	1161 (15.9)	86 (16.0)			
Missing	88	80	8			
HRT				0.228		
No	7224 (91.3)	6718 (91.2)	506 (92.7)			
Yes	691 (8.7)	651 (8.8)	40 (7.3)			
**Clinical features** ^‡^						
TOP-T				<0.001		
No	2488 (31.4)	2418 (32.8)	70 (12.8)		1.00 (reference)	
Yes	5427 (68.6)	4951 (67.2)	476 (87.2)		4.85 (3.62, 6.52)	<0.001
TOP-O				<0.001		
No	6576 (83.1)	6170 (83.7)	406 (74.4)		1.00 (reference)	
Yes	1339 (16.9)	1199 (16.3)	140 (25.6)		1.73 (1.34, 2.23)	<0.001
TOP-P				<0.001		
No	5281 (66.7)	4808 (65.2)	473 (86.6)		1.00 (reference)	
Yes	2634 (33.3)	2561 (34.8)	73 (13.4)		0.46 (0.35, 0.61)	<0.001

Values in parentheses are percentages unless indicated otherwise;

*values in parentheses are 95 per cent c.i.;

^†^values are median (i.q.r.).

^‡^Clinical features were extracted from primary care routine and urgent referrals, and then stratified into the TOP terms: T, tumour; O, observation; P, somatosensory perception (see *Table 2*). HRT, hormone replacement therapy.

^§^Multivariable model: overall model fit *P* < 0.001, Nagelkerke *R*^2^ = 0.103, Hosmer and Lemeshow test *P* = 0.505, area under the receiver operating characteristic (ROC) curve 0.824 (s.e. 0.010).

### Overall Accuracy 

**Fig. 2:**
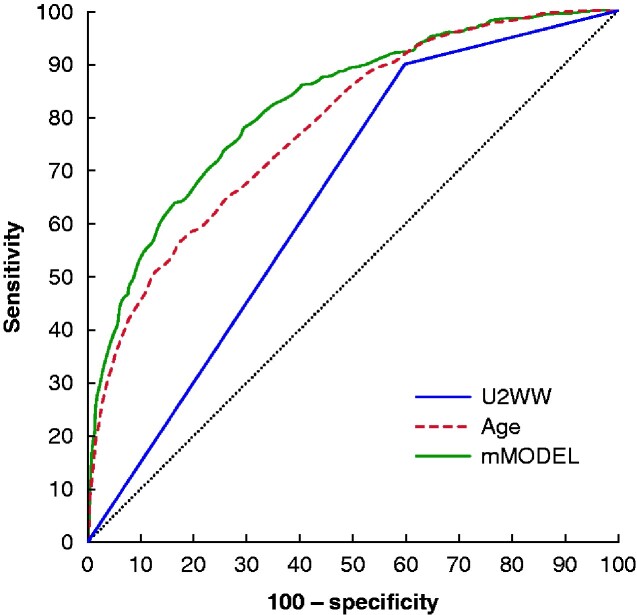
Comparison of receiver operating characteristic (ROC) curves for urgent 2-week referral for suspected breast cancer criteria, patient age alone, and a multivariable model with respect to discrimination of cancer *versus* no cancer U2WW, urgent 2-week referral for suspected breast cancer criteria; mMODEL, multivariable model. Area under the ROC curve (AUC): 0.651 (95 per cent c.i. 0.641 to 0.662) for U2WW *versus* 0.784 (0.773 to 0.791) for age alone *versus* 0.824 (0.815 to 0.832) for mMODEL; *P* <0.001 for all comparisons).

The U2WW had a sensitivity of 89.9 (95 per cent c.i. 87.1 to 92.3) per cent, specificity of 40.5 (39.4 to 41.6) per cent, PPV of 10.1 (9.8 to 10.4) per cent, FOR of 1.8 (1.4 to 2.4) per cent, and an AUC of 0.651 (0.641 to 0.662), indicating better discrimination than random assignment (null hypothesis AUC 0.500). Age alone performed significantly better than the U2WW (AUC 0.784, 0.773 to 0.791), and the multivariable model performed better than age (AUC 0.824, 0.815 to 0.832), across all cut-off values (*Fig. 2*).

### Accuracy considering cost assumptions and optimal referral thresholds

*Table 4* shows the ORT for each model at each of the eight cost assumptions, the proportion of the total that would be referred urgently at the ORT, accuracy parameters, net cost, and percentage cost increment or decrement. The multivariable model and age alone were generally better than the U2WW at meeting the cost challenges posed by the hypothetical cost assumptions (lower net cost and/or more accurate), and the multivariable model offered a further advantage over age.

**Table 4:** Comparison of the referral models considering the cost assumptions

	ORT	**Urgent** ^*^ **/** **total**	Sensitivity (%)	Specificity (%)	PPV (%)	FOR (%)	AUC	*P* ^§^	*P* ^¶^	**Cost** ^†^	**ΔCost (%)** ^‡^ **^§^**	**ΔCost (%)**‡**^¶^**
**Cost assumption: FNc : FPc=150 : 1, TPc : TNc=−1 :−1**												
U2WW	Urgent	7915 (100)	100 (99.3, 100)	0.0 (0.0, 0.0)	6.9 (6.9, 6.9)	n.a.	0.500 (0.489, 0.511)	Ref	–	1.16	Ref	–
Age	≥29 years	6934 (87.6)	99.6 (98.7, 100)	13.3 (12.5, 14.1)	7.8 (7.8, 7.9)	0.2 (0.1, 0.8)	0.565 (0.554, 0.576)	<0.001	Ref	0.65	−44	Ref
mMODEL	≥1.0%	6248 (79.9)	98.7 (97.3, 9.5)	21.7 (20.7, 22.6)	8.3 (8.2, 8.4)	0.4 (0.2, 1.0)	0.601 (0.590, 0.612)	<0.001	<0.001	0.60	−48	−8
**Cost assumption: FNc : FPc=100 : 1, TPc : TNc=−1 :−1**												
U2WW	Referral criteria	4877 (61.6)	89.9 (87.1, 92.3)	40.5 (39.4, 41.6)	10.1 (9.8, 10.4)	1.8 (1.4, 2.4)	0.651 (0.641, 0.662)	Ref	–	0.81	Ref	–
Age	≥40 years	5387 (68.1)	95.4 (93.3, 97.0)	34.0 (32.9, 35.1)	9.7 (9.5, 9.9)	1.0 (0.7, 1.5)	0.647 (0.641, 0.663)	0.555	Ref	0.55	−32	Ref
mMODEL	≥1.8%	5232 (66.9)	95.3 (93.1, 96.9)	35.2 (34.1, 36.3)	9.6 (9.4, 9.8)	1.0 (0.6, 1.4)	0.652 (0.641, 0.663)	0.945	0.444	0.53	−34	−4
**Cost assumption: FNc : FPc=50 : 1, TPc : TNc=−1 :−1**												
U2WW	Referral criteria	4877 (61.6)	89.9 (87.1, 92.3)	40.5 (39.4, 41.6)	10.1 (9.8, 10.4)	1.8 (1.4, 2.4)	0.651 (0.641, 0.662)	Ref	–	0.46	Ref	–
Age	≥44 years	4504 (56.9)	89.6 (86.7, 92.0)	45.5 (44.4, 46.7)	10.9 (10.5, 11.2)	1.7 (1.3, 2.2)	0.675 (0.665, 0.686)	0.020	Ref	0.38	−17	Ref
mMODEL	≥3.9%	3393 (43.4)	85.9 (82.7, 88.8)	59.7 (58.6, 60.8)	13.3 (12.8, 13.9)	1.7 (1.3, 2.1)	0.728 (0.718, 0.738)	<0.001	<0.001	0.23	−50	−39
**Cost assumption: FNc : FPc=1 : 1, TPc : TNc=−1 :−1**												
U2WW	C&B	0 (0)	0.0 (0.0, 0.7)	100 (99.9, 100)	n.a.	6.9 (6.4, 7.5)	0.500 (0.489, 0.511)	Ref	–	−0.86	Ref	–
Age	≥88 years	81 (1.0)	9.7 (7.4, 12.5)	99.6 (99.5, 99.7)	65.4 (54.7, 74.8)	6.3 (5.8, 6.9)	0.548 (0.537, 0.559)	<0.001	Ref	−0.87	−1	Ref
mMODEL	≥44.8%	144 (1.8)	18.6 (15.4, 22.2)	99.4 (99.2, 99.5)	68.1 (60.3, 74.9)	5.6 (5.1, 6.1)	0.590 (0.579, 0.601)	<0.001	<0.001	−0.88	−2	−1
**Cost assumption: FNc : FPc=150 : 1, TPc : TNc=−10 :−1**												
U2WW	Urgent	7915 (100)	100 (99.3, 100)	0.0 (0.0, 0.0)	6.9 (6.9, 6.9)	n.a.	0.500 (0.489, 0.511)	Ref	–	0.24	Ref	–
Age	≥29 years	6934 (87.6)	99.6 (98.7, 100)	13.3 (12.5, 14.1)	7.8 (7.8, 7.9)	0.2 (0.1, 0.8)	0.565 (0.554, 0.576)	<0.001	Ref	0.03	−88	Ref
mMODEL	≥1.0%	6248 (79.9)	98.7 (97.3, 99.5)	21.7 (20.7, 22.6)	8.3 (8.2, 8.4)	0.4 (0.2, 1.0)	0.601 (0.590, 0.612)	<0.001	<0.001	0.00	−100	−100
**Cost assumption: FNc : FPc=100 : 1, TPc : TNc=−10 :−1**												
U2WW	Urgent	7915 (100)	100 (99.3, 100)	0.0 (0.0, 0.0)	6.9 (6.9, 6.9)	n.a.	0.500 (0.489, 0.511)	Ref	–	0.24	Ref	–
Age	≥40 years	5387 (68.1)	95.4 (93.3, 97.0)	34.0 (32.9, 35.1)	9.4 (9.2, 9.6)	1.0 (0.7, 1.5)	0.647 (0.641, 0.663)	<0.001	Ref	−0.04	−117	Ref
mMODEL	≥1.1%	6162 (78.8)	98.5 (97.0, 99.3)	22.7 (21.7, 23.6)	8.4 (8.3, 8.5)	0.5 (0.2, 1.0)	0.606 (0.595, 0.616)	<0.001	<0.001	−0.05	−121	−25
**Cost assumption: FNc : FPc=50 : 1, TPc : TNc=−10 :−1**												
U2WW	Referral criteria	4877 (61.6)	89.9 (87.1, 92.3)	40.5 (39.4, 41.6)	10.1 (9.8, 10.4)	1.8 (1.4, 2.4)	0.651 (0.641, 0.662)	Ref	–	−0.10	Ref	–
Age	≥40 years	5387 (68.1)	95.4 (93.3, 97.0)	34.0 (32.9, 35.1)	9.7 (9.5, 9.9)	1.0 (0.7, 1.5)	0.647 (0.641, 0.663)	0.555	Ref	−0.20	−100	Ref
mMODEL	≥3.9%	3393 (43.4)	85.9 (82.7, 88.8)	59.7 (58.6, 60.8)	13.3 12.8, 13.9)	1.7 (1.3, 2.1)	0.728 (0.718, 0.738)	<0.001	<0.001	−0.29	−190	−45
**Cost assumption: FNc : FPc=1 : 1, TPc/TNc=−10 :−1**												
U2WW	C&B	0 (0)	0.0 (0.0, 0.7)	100 (99.9, 100)	n.a.	6.9 (6.4, 7.5)	0.500 (0.489, 0.511)	Ref	–	−0.86	Ref	–
Age	≥71 years	798 (10.1)	42.5 (38.3, 46.8)	92.3 (91.7, 92.9)	29.1 (26.6, 31.7)	4.4 (4.0, 4.9)	0.672 (0.662, 0.683)	<0.001	Ref	−1.04	−21	Ref
mMODEL	≥19.3%	670 (8.6)	44.7 (40.4, 49.0)	94.0 (93.5, 94.6)	95.9 (95.6, 96.2)	4.7 (3.6, 4.6)	0.694 (0.683, 0.704)	<0.001	0.010	−1.09	−27	−5

Values in parentheses are 95 per cent c.i. unless indicated otherwise;

*values in parentheses are percentages of the total (7915 for urgent 2-week referral for suspected breast cancer criteria (U2WW) and age, and 7821 for the multivariable model (mMODEL).

^†^Average cost given by: (TPc × TPp) + (TNc × TNp) + (FPc × FPp) + (FNc × FNp), where TPp is the proportion of true positives in the sample, FNp is the proportion of false negatives in the sample, and so on; benefits expressed as negative costs.

^‡^ΔCost is given by (average cost of the model − average cost of reference model)/cost of reference model) × 100 per cent. ORT, optimal referral threshold; PPV, positive predictive value; FOR, false omission rate; AUC, area under the receiver operating characteristic (ROC) curve of the respective model at the optimal referral criterion; FPc, cost of a false-positive (urgent) referral; FNc, cost of a false-negative (choose and book referral route; C&B) referral; TPc, cost of a true-positive (urgent) referral; TNc, cost of a true-negative (C&B) referral; n.a., calculation cannot be performed because the values entered include one or more instances of zero.

^§^U2WW (reference category) *versus* age and mMODEL;

^¶^age (reference category) *versus* mMODEL.

## Discussion

In this study, performance of the U2WW was compared with the multivariable model and age alone in discriminating cancer *versus* no cancer. The U2WW had high sensitivity (89.9 per cent), but at the expense of poor specificity (40.5 per cent); age alone outperformed U2WW, and the multivariable model offered a further advantage.

Univariable analysis of the 10 selected and potentially predictive factors for a diagnosis of breast cancer in the breast clinic following primary care referral is flawed owing to confounding influences; multivariable analysis mitigated this, and showed that personal history of breast cancer, menopause, breastfeeding, first- or second-degree relative with breast cancer, and hormone replacement therapy (HRT) were not influential. There is no doubt that such factors are—or are approximations for—epidemiological risks^19^, but the referral process rendered them non-contributory. It is possible that some of these histories amplified awareness and concern, and promoted referral.

After multivariable adjustment, the multivariable model was more accurate than U2WW (AUC 0.824 *versus* 0.651 respectively; *P* < 0.001) and age alone (AUC 0.842 *versus* 0.784; *P* < 0.001). The inferior performance of the U2WW is multifactorial. First, although each of the U2WW referral criteria might be known individually to predict a risk of breast cancer of 3 per cent or more (or 5 per cent according to the old criteria), the risk associated with each is not necessarily similar. However, the criteria were given equal weight; for example, nipple discharge in a 50-year-old woman weighed equally to an unexplained lump in a 49-year-old. Second, although most of the U2WW criteria carry an age tag, the superior performance of age, as a single referral criterion, suggests that age was not weighted adequately. Third, the U2WW does not consider negative predictors of breast cancer such as TOP-P or gravidity, which were shown in this study to be influential. Finally, although referral is indicated by clinical findings and acumen, the process is complex and multifactorial^20^; modification by patient demand, lack of direct access by GP to breast investigations, and pressure from governance bodies are unavoidable influences. These inputs constitute selection pressures for and on the epidemiological risks, and perhaps the clinical symptoms and signs of breast cancer. The multivariable model is less prone to these biases, and age alone is immune.

These shortcomings of the U2WW should not come as a surprise. The U2WW criteria were extracted from only five methodologically heterogeneous and biased studies^21–25^, such that meta-analysis was not possible and the developers of the guidance themselves expressed their concerns regarding their validity and applicability^10^.

ROC curve analysis and the associated AUC of the models give an overall picture of the models’ behaviour across all cut-off values. As continuous metric parameters, age and the probabilities of breast cancer produced by the multivariable model must be dichotomized at specific cut-off values that discriminate high *versus* low risk if they are to operate a dual-route referral system, and in the interest of fair comparison with the binary U2WW. Conceptually, the ORT is the ideal cut-off value that strikes the best balance between sensitivity and specificity, resulting in the lowest net cost of any predictive model or diagnostic test. Therefore, referral models are best compared at their respective ORTs, an approach that requires summation of the costs and benefits (health plus financial) associated with each of the possible false and true outcomes (FN, FP, TP and TN) on a common scale. Such an endeavour requires scientific, public and governmental inputs, and is beyond the scope of this study. Therefore, several cost and benefit assumptions were made that would result in a wide range of ORT values for each of the models under evaluation in order to demonstrate their behaviour under various cost pressures.

In a system with complex health and political considerations and finite resources, the cut-off value for an urgent referral should not be chosen arbitrarily. In June 2015, new criteria were chosen such that they corresponded to a risk of breast cancer of 3 per cent or above, rather than 5 per cent or more as in the older version. The assumption was that lowering the threshold for urgent referrals would significantly improve the detection rate (sensitivity) and reduce the proportion of women with breast cancer referred through C&B (FOR) with no significant attrition of specificity. However, subanalysis in the present study found no significant difference in sensitivity and FOR, and a significantly lower specificity using the new criteria.

Use of a referral process that employs two pathways (urgent *versus* routine) or more can be justified only if it meets the existing cost challenge (health and financial) at lower net cost than simply referring all patients urgently (or routinely). In the present study, when the perceived cost of referring a symptomatic patient erroneously through the routine (C&B) route (FNc) relative to the cost of referring a patient erroneously through the urgent route (FPc) was very high (150 : 1), the U2WW referral criteria became redundant, as the hypothesized cost requirements were best met by referring all patients urgently, as indicated by the ORT. Age alone and the multivariable model, however, continued to meet the cost challenge whilst operating dual routes in which urgently referred patients comprised 87.6 and 79.9 per cent of the total respectively. In the same cost climate, age at the 29 years cut-off was more accurate than U2WW, and the multivariable model, at 1.0 per cent probability of breast cancer, offered a further advantage. Both age and the multivariable model were associated with a health cost saving ranging from 44 to 88 per cent, and 48 to 100 per cent respectively. Similarly, when FNc was very low and equal to FPc, the lowest cost of the system was achieved by referring all patients through C&B, decimating the urgent route, unlike for age and the multivariable model. That said, it remains unknown whether, or what, underlying costs and benefits were considered when the U2WW criteria and process were rolled out, and the assumption that the U2WW is safer and less costly than referring all patients to secondary care indiscriminately remains unfounded.

The multivariable model performed significantly better than age alone, but the difference was modest when various ORTs were considered. This is probably because the retrospective grouping of reported symptoms and signs into the TOP terms may have diluted their influence. The TOP system facilitated the evaluation of the data, but did not allow for nuances in the clinical findings. An example is that a fixed hard mass is a ‘tumour’ (TOP-T), but so is nodularity, which can have very different implications. The study’s clinical database allows for more granularity and complexity within the clinical symptomatology criteria, but the simplicity of the TOP approach is convenient for evaluating high *versus* low risk. The U2WW criteria include ambiguous or subjective referral terms such as ‘unexplained lump’, ‘changes of concern’ and ‘skin changes that suggest cancer’, which may have contributed to referral biases. The TOP terms, on the other hand, are clear, objective and reproducible. Applying the TOP terms, or any other similarly unambiguous and easy-to-use system, at the level of primary care can improve the accuracy of multivariable models. In addition, an evaluation of all known risk and protective factors^19^ for breast cancer was not possible. Consequently, factors that were obtainable and regarded as evidentially pertinent^26^^,^^27^ were chosen; other combinations could be advantageous. Finally, the approach to cleaning and stratification of the data meant that these factors may not reflect the intensity of the association with breast cancer adequately (for example, HRT was for any duration, type or patient age, so were gravidity and breastfeeding). However, such a pragmatic approach simplified statistical analysis and is more appropriately suited for the busy primary care setting. Refinements would be required if a multivariable model were to be considered, and could be achieved by larger-scale prospective data collection.

The GP needs only to make a decision as to whether a woman requires referral at all; subsequently, determination of the route for referral is streamlined in either primary or secondary care, according to the agreed breast cancer probability threshold. Predictive multivariable models that summate negative and positive predictors, and use simple and reproducible clinical terms, may offer an additional advantage and allow multitier stratification of risk for better utilization of resources, unlike U2WW. The U2WW criteria lack scientific validation, and it is time to consider alternatives. Indeed, it may be time for the gatekeeper role of primary care to be reconsidered^9^. The U2WW process consists of two parts: risk prediction for breast cancer, on which access to specialist care is based. Such a system is bound to be challenging for primary care, which will guard against undersuspicion of breast cancer. If the outcome of the referral process from primary care is predictable by a regression equation including pertinent patient demographics and simple clinical terms, the same may be possible for patients themselves: online, by phone, or in secondary care walk-through clinics.

## Funding

The study was sponsored by the University Hospitals Plymouth NHS Trust which also funded the publication fees.

The authors also received funding from the Primrose Foundation (UK registered charity; number 1064277) to pay for statistical analysis.
